# P-589. Investigation of a Whooping Cough Outbreak in Grand Traverse County, Michigan—July 2024-December 2024

**DOI:** 10.1093/ofid/ofaf695.803

**Published:** 2026-01-11

**Authors:** Tanner Porter, Kyle Muchez, Jacalyn M Money-Bruno, Emily Maas, Maria Santana-Garces, Rebekka Pittsley, Ahlam M Rahimee, Kyle G Crooker, Seema Joshi, Marcus Zervos, Yasmeen Mann, Najibah K Rehman

**Affiliations:** Grand Traverse County Health Department, Traverse City, Michigan; Grand Traverse County Health Department, Traverse City, Michigan; Grand Traverse County Health Department, Traverse City, Michigan; Grand Traverse County Health Department, Traverse City, Michigan; Henry Ford Health, Livonia, MI; Michigan State University College of Human Medicine, East Lansing, Michigan; Michigan State University College of Human Medicine, East Lansing, Michigan; Henry Ford Hospital, Detroit, MI; Henry Ford Hospital, Detroit, MI; Henry Ford Hospital, Detroit, MI; Henry Ford Hospital, Detroit, MI; Henry Ford Health System, Detroit, MI

## Abstract

**Background:**

Global surges attributed to whooping cough, caused by *Bordetella pertussis*, are widespread. Maternal Tdap demonstrates success in preventing infant morbidity and mortality, however, cases predominate in adolescents. Unvaccinated individuals are at risk of infection. However, further exploration is warranted regarding waning immunity. This investigation outlines a whooping cough outbreak.

**Figure:**
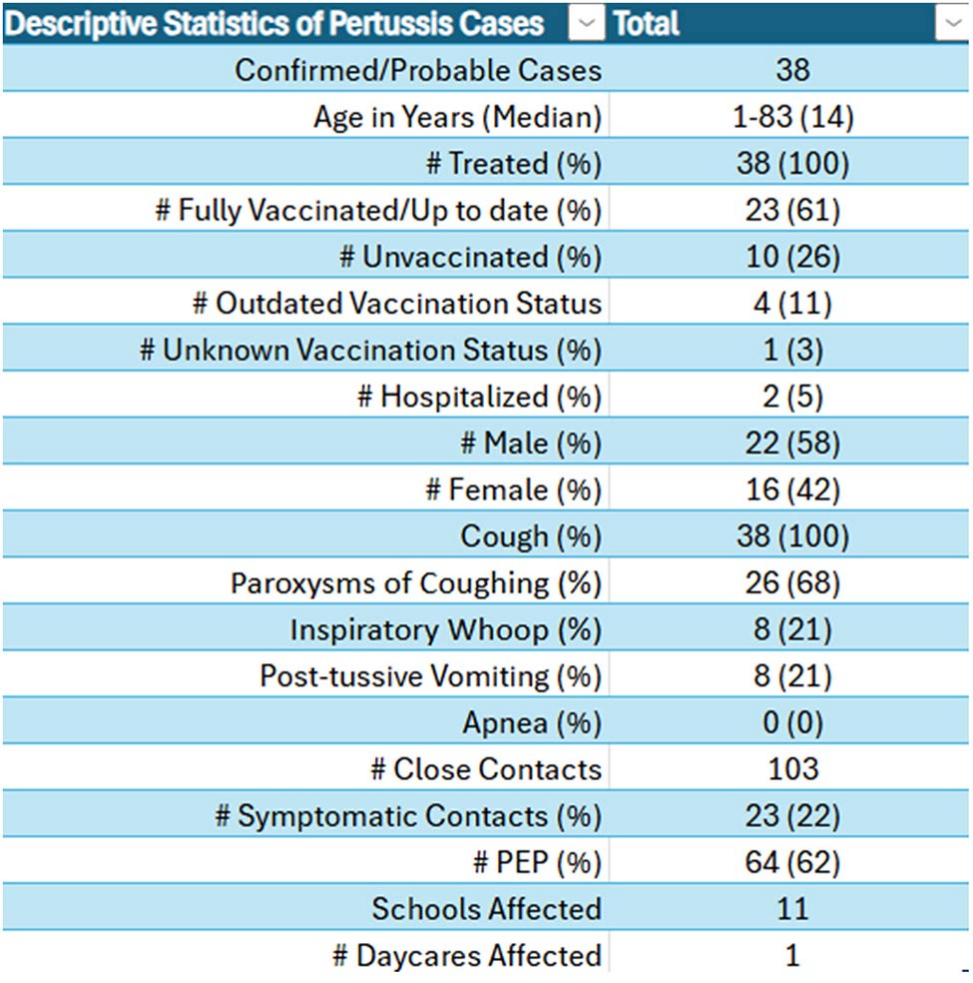

**Figure:**
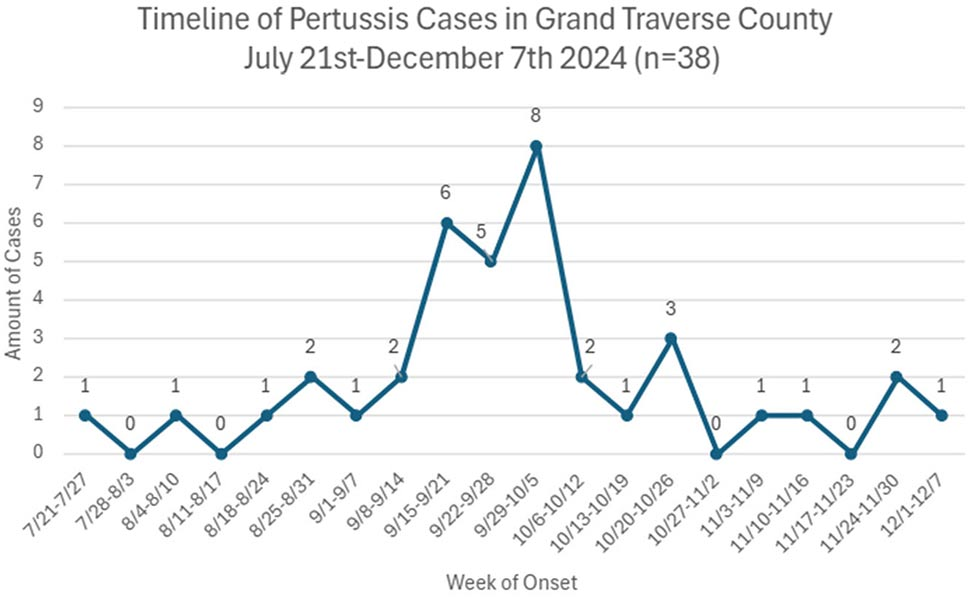

**Methods:**

38 cases were identified from July 2024-December 2024 through health facilities, schools, families and the Michigan Disease Surveillance System (MDSS). Cases were investigated using the MDSS pertussis case investigation form for demographics, symptoms, hospitalization, vaccination, treatment and contact tracing. Close contacts were notified for mitigation. Immunizations and waiver data was obtained through the Michigan Care Improvement Registry (MCIR).

**Figure:**
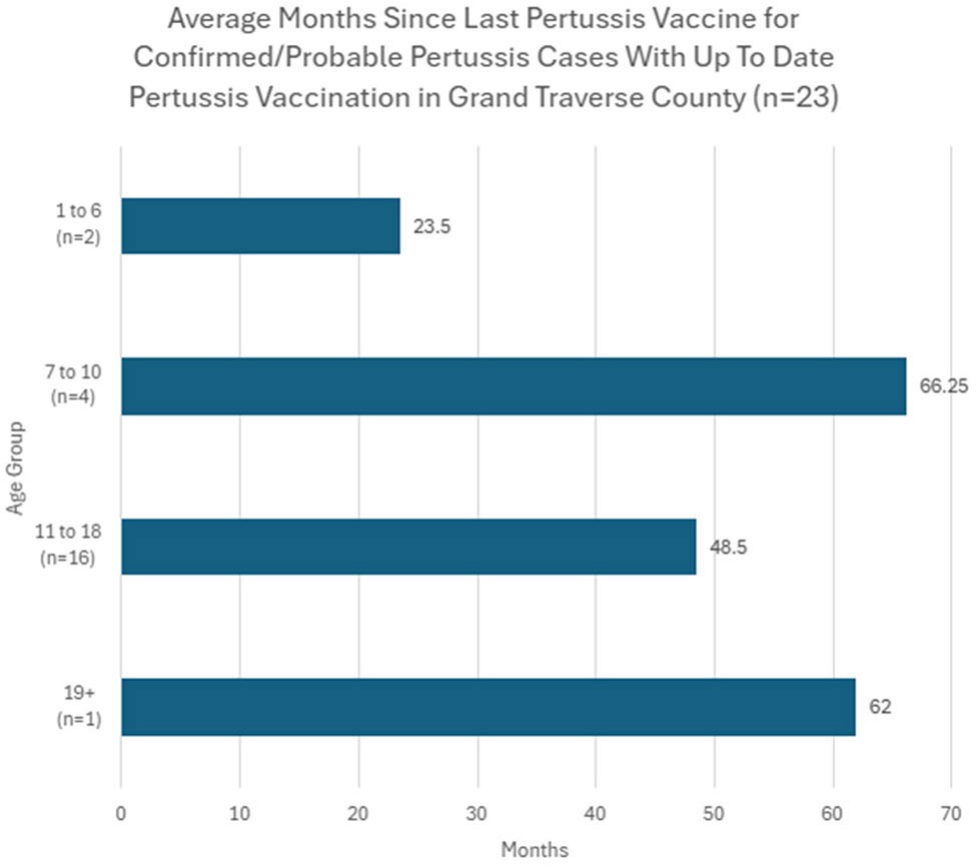

**Figure:**
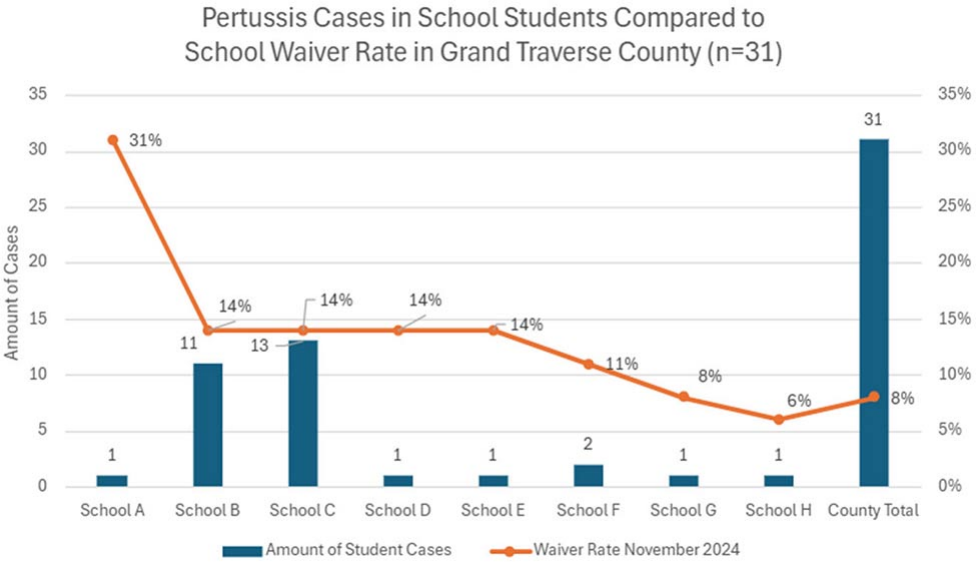

**Results:**

38 cases were identified, 1-84 years old (median 14). 61% (n=23) of infections occurred in 11-18 years, 13% (n=5) each in 1-6, 7-10, and 19+ age cohorts. 61% were fully vaccinated (n=23), 26% unvaccinated (n=10), 14% outdated/unknown status (n=5). Average time since last dose among fully vaccinated was 23.5 months in 1-6 years (n=2), 66.25 in 7-10 (n=4), 48.5 in 11-18 (n=16) and 62 in 19+(n=1) cohorts. Primary symptoms were cough (100%), paroxysms of cough (68%), inspiratory whoop (21%), post-tussive vomiting (21%). 100% received treatment, 2 were hospitalized, no deaths and no cases in < 1-year olds. 11 schools and 1 daycare were affected. Among the top two schools with students impacted, immunization waiver percentages were 14% each, compared to county waivers of 8%.

**Conclusion:**

Two major factors are seen in our outbreak: 1) prolonged time since last dose for fully vaccinated individuals, and 2) non/under vaccinated people. Immunizations waivers place unvaccinated at high risk for infection. Efforts to encourage vaccination continue. However, the number of cases in fully vaccinated youth underscore the need to investigate waning immunity. The economic impact on individuals, families and society causing classroom shutdowns, sick time, lost wages, and over-utilization of healthcare and personnel resources persist. Consideration of boosters after adolescent Tdap or improved vaccine development are necessary.

**Disclosures:**

Seema Joshi, MD, Cephied: Grant/Research Support Marcus Zervos, MD, merck: Honoraria

